# Laboratory investigations in the diagnosis and follow-up of GH-related disorders

**DOI:** 10.20945/2359-3997000000192

**Published:** 2019-11-01

**Authors:** Katharina Schilbach, Martin Bidlingmaier

**Affiliations:** 1 Medizinische Klinik und Poliklinik IV Klinikum der Universität München Munich Germany Medizinische Klinik und Poliklinik IV, Klinikum der Universität München, Munich, Germany

**Keywords:** Growth hormone, insulin-like growth factor I, binding proteins, growth hormone deficiency, acromegaly

## Abstract

In addition to auxiological, clinical and metabolic features measurements of growth hormone (GH) and insulin-like growth factor I (IGF-I) complement our tools in diagnosis and follow-up of GH-related disorders. While comparably robust during the pre-analytical phase, measurement and interpretation of concentrations of both hormones can be challenging due to analytical issues and biological confounders. Assay methods differ in terms of antibody specificity, interference from binding proteins, reference preparations and sensitivity. GH assays have different specificity towards different GH-isoforms (e.g. 20 kDa GH, placental GH) and interference from the GH antagonist Pegvisomant. The efficacy to prevent binding protein interference is most important in IGF-I assays. Methodological differences between assays require that reference intervals and diagnostic cut-offs are assay-specific. Among biological variables, pubertal development and age are most relevant for IGF-I, making detailed reference intervals mandatory for interpretation. GH has pulsatile secretion and short half-life. Its concentration is modified by acute factors such as stress, exercise and sleep, but also by intake of oral estrogens and anthropometric factors (e.g. BMI). Other GH dependent biomarkers such as free IGF-I, IGF binding protein 3 (IGFBP 3) and acid labile subunit (ALS) have been proposed. Their concentrations largely mirror the information obtained through measurement of IGF-I, but their measurement can be helpful in particular situations. In this review, we describe the evolution of analytical methods to measure biomarkers of GH action, the impact of the methodological changes on laboratory results and the need to include biological variables in their interpretation. Arch Endocrinol Metab. 2019;63(6):618-29

## INTRODUCTION

In all growth hormone (GH) related disorders – GH deficiency (GHD), GH insensitivity and GH excess – GH and insulin-like growth factor-I (IGF-I) are the most important biomarkers used for diagnosis and during follow-up. Other parameters, such as insulin-like growth factor binding protein 3 (IGFBP 3) and acid labile subunit (ALS), can be useful in particular situations, but overall their diagnostic relevance is limited ( 1 , 2 ). Although GH and IGF-I are widely used, both biomarkers have shortcomings due to particularities of the analytical process itself, but also due to difficulties in the interpretation of the results. Discrepancies between the results from measurements of GH and IGF-I concentrations have been reported repeatedly ( 3 ) and can lead to problems in clinical management.

Throughout the last decades, GH and IGF-I assays evolved. Generally, assays have become more sensitive and specific. Nevertheless, significant differences in the results obtained from measurements by different laboratories or with assays from different manufacturers still are common ( 4 ). In addition to analytical issues, a wide spectrum of endogenous and pharmacological factors influence circulating concentrations of the hormones and need to be taken into account.

This review focusses on the analytical and interpretative aspects related to GH and IGF-I concentrations. We also describe less frequently used GH-related biomarkers. Suitability of specific stimulation and suppression tests and the respective diagnostic cut-offs have been extensively discussed elsewhere, and are not the primary focus of this article ( 5 - 8 ).

## GROWTH HORMONE

### GH molecule

GH is a polypeptide hormone and a cytokine of the growth factor superfamily. GH is mainly expressed in somatotropic cells of the pituitary gland. It is secreted into the blood stream and mediates its effects via dimerized GH receptors in many tissues. GH in circulation consists of a variety of different isoforms, fragments and molecular complexes (homo- and hetero-dimers and oligomers) ( 9 ). With over 90%, the 22kDa isoform ( 22 ,129 Da) is the most abundant isoform, and best reflects total pituitary GH secretion ( 10 ). Therefore, current guidelines request that modern GH assays should be designed to specifically measure this isoform ( 4 , 11 ). The second most abundant isoform is the 20kDa isoform ( 20 ,274 Da), although the biological significance of this isoform has not yet been fully understood ( 9 ). In addition, other isoforms with minor chemical modifications exist ( 10 ). In circulation, the different isoforms aggregate to some extent, thereby forming dimers and multimers. One particular GH isoform occurs only in females during pregnancy. It is synthesized and secreted by the placenta and therefore termed “placental growth hormone” (GH-V). Recently, the spectrum of GH isoforms was further increased by the invention of a mutated GH molecule with antagonistic properties. This artificial isoform today is known as the GH antagonist pegvisomant and used in the treatment of acromegaly. It binds to the GH receptor but inhibits signal transduction and hence IGF-I release ( 12 , 13 ).

### Technical aspects of GH measurements

To measure GH concentrations for clinical routine purposes, assays from different commercially sources as well as some in-house methods are being used. Most of the assays still recognize a broader spectrum of GH isoforms or have unknown isoform specificity. However, some of today’s routine assays have already incorporated recent recommendations and specifically detect the 22kDa isoform only ( 4 , 11 ). As indicated above, current evidence suggests that this isoform represents total pituitary GH secretion. Although of scientific interest, available studies on specific measurement of isoforms other than 22kD GH did not reveal additional diagnostic value in clinical routine situations.

Detection of GH activity in humans was first described in 1955, while the first GH immunoassay was reported in 1961 ( 14 , 15 ). However, the molecular structure of human GH wasn’t discovered until the 1970^th^ ( 16 ). Over the past decades, GH assays evolved from relatively unspecific radioimmunoassays to modern, highly sensitive chemiluminescence immunoassays. Specificity increased by the use of monoclonal antibodies rather than polyclonal antisera ( 11 ). Most older assays had recognized a spectrum of different GH isoforms together with their homo- and heterodimers and – multimers, while monoclonal antibodies recognize a defined epitope on the surface of the GH molecule and therefore tend to only pick a narrow spectrum of all the GH molecules in circulation. This is part of the explanation why GH concentrations as measured by newer assays tend to be lower than those measured by assays which were available 20 years ago. It is also important to keep in mind that other GH isoforms such as the GH antagonist pegvisomant or GH-V can significantly cross-react with GH assays. Less specific assays might also cross-react with closely related molecules such as prolactin or placental lactogen. Currently, there is only one automated GH immunoassay that does not have cross-reactivity with all of those molecules ( 13 ). Another factor potentially affecting reported GH concentrations is interference from growth hormone binding protein (GHBP). In circulation, approximately 50% of GH is bound to GHBP, and reliable GH immunoassays should ensure that relevant epitopes are not hidden through GHBP binding ( 11 ).

Alongside changes in assay specificity, there was also an evolution of the standard preparations used to calibrate the assays: Originally, only cadaveric GH extracted from pituitaries was available. These preparations, including international standards 66/217 and 80/505, were purified to some degree, but still consisted of a mixture of isoforms. When recombinant GH became available, the international reference preparation IRP 88/624 was introduced ( 4 , 17 ). Since this preparation consisted solely of pure 22kD GH, the signal generated from this calibrator compared to the pituitary extracts was stronger in assays preferentially detecting this isoform, while it was weaker in assays preferentially recognizing other isoforms. Consequently, depending on the isoform specificity of the assay used, reported GH concentrations for clinical samples changed. Because reference preparations of recombinant origin can be much better standardized than pituitary extracts, and because they allow traceability to mass concentrations rather than arbitrary units, guidelines strongly recommend the use of such recombinant preparations ( 4 ). Today the most common preparation used to calibrate GH assays is the latest recombinant IRP 98/574 ( Table 1 ). The uniform use of this preparation has removed some, though not all, of the differences in GH concentrations reported from different GH assays ( 18 ).

**Table 1:** Technical characteristics of some widely used, automated GH and IGF-I assays

	Assay platform	Assay type	range	Sensitivtiy (LoQ)	Sensitivity (LoD)	Specificity/Interference	Standard
**GH (ng/mL)**	IDS iSYS	CLIA	0.04-100	0.04	0.015	No cross-reactivity	WHO IS 98/574
	Diasorin Liaison	CLIA	0.05-80	0.05	0.05	Pegvisomant (false positive), 20kDa hGH, placental GH, human placental lactogen	
	Roche Cobas	ECLIA	0.03-50	0.05	0.03	Pegvisomant (effect not specified), 20 kDa hGH, placental GH	
	Siemens Immulite	CLIA	0.1-40	0.05	0.01	Pegvisomant (false negative), 20kDa hGH, placental GH, human placental lactogen	
IGF-I (ng/mL*)	IDS iSYS	CLIA	10-1200	8.8	4.4		WHO IS 02/254
	Diasorin Liaison	CLIA	10-1000	10	3		
	Roche Cobas	ECLIA	7-1600	15	7		
	Siemens Immulite	CLIA	20-1600	19.7	14.4		

GH: growth hormone; IGF-I: insulin-like growth factor I; CLIA: chemiluminescence immunoassay; ECLIA: electrochemiluminescence immunoassay; LoQ: limit of quantification; LoD: limit of detection; WHO IS: World Health Organization International Standard;

*Conversion factor IGF-I to nmol/Lng/mL: x 0.131.

GH assays also developed with respect to sensitivity. While earlier assays could not detect GH concentrations below 2.5-5.0 µg/L, the introduction of high affinity monoclonal antibodies and new labelling technologies improved sensitivity down to 1 µg/L or below. Ultrasensitive GH assays with a sensitivities as low as 0.002 ug/L were first described in the 1990s ( 19 ). While diagnostic relevance of measuring such low GH concentrations remains to be demonstrated, current literature agrees that reliable assessment of concentrations well below 1 µg/L is required when assessing suppression of GH in patients with acromegaly. Accordingly, guidelines recommend to only use assays with proven lower limits of quantification (LoQ) at 0.05 ug/L. It is important for laboratories that this sensitivity can be achieved on a daily routine basis and not only in research assays in specific settings. Notably, sufficient reproducibility is key at the low end, and should not exceed 20% ( 4 ).

All these analytical factors are far more important determinants of reported GH concentrations than classical preanalytical factors. While some peptide hormones are very sensitive to temperature and storage time, GH is a fairly stable molecule, making preanalytical sample handling as well as storage conditions rather uncomplicated. Long-term sample storage for 5 years is possible at -20°C, and even storage for more than 10 years at -80°C was not associated with any change in GH concentrations ( 20 ).

### Impact of analytical methods on GH reference ranges and cut-offs

GH is secreted in a pulsatile manner with age- and gender-specific differences in pulse frequency, peak pulsatility and circadian pattern of pulses ( 21 ). Therefore, random GH has limited diagnostic value and its use is not recommended for diagnosis or follow-up of GHD or acromegaly ( 5 - 7 ). Depending on the suspected diagnosis, stimulation tests (e.g. insulin tolerance test, combined GH releasing hormone arginine test, glucagon test, clonidine test, macimorelin test) or a suppression test (oral glucose tolerance test (OGTT)) and GH day profiles are used to evaluate GH secretory status ( 5 - 7 , 22 ). Since absolute GH concentrations reported by different assays or laboratories for an identical sample can differ significantly for the technical reasons described above, any cut-offs used for interpretation of the results of dynamic tests ideally should be assay-specific. The evolution of both, specificity and sensitivity of the analytical methods to measure GH is the main reason why there is a continuous decline in the GH cut-offs proposed by guidelines. In GHD, earlier recommendations referred to concentrations above 10 µg/L, while many of the newer recommendations propose cut-offs at 7 μg/L or lower ( 23 ). Similarly, GH concentrations after glucose suppression stated in guidelines on diagnosis and management of acromegaly droppeded from < 5 µg/L and < 2.5 µg/L to < 0.4 µg/L ( 8 , 24 , 25 ). Using a sensitive, modern automated assay, we recently demonstrated that normal GH nadir concentrations in obese healthy males might even be lower than that ( 26 ).

**Figure 1:**
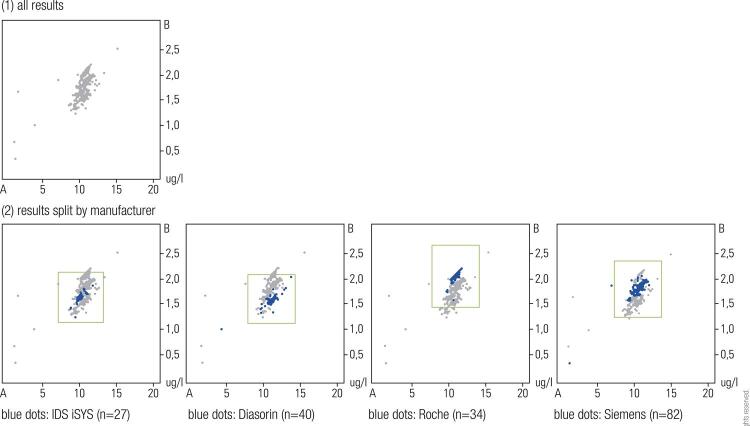
Measurement of GH in the same two samples A and B by different laboratories (n=208). Reported concentrations vary by more than 100% ( 1 ). The same results split by manufacturer reveal systematic differences between the respective assay methods. Three of the automated assays are shown as an example ( 2 ). Results taken from the External Quality Assessment Scheme 4/2017 organized by Reference Institute for Bioanalytics (RfB, Bonn, Germany), one of the two German proficiency testing organizations. More results can be accessed at http://www.rfb.bio

Given the technical differences between GH measurements conducted in different labs by different methods, for consistence during monitoring it would be desirable to have all samples from a patient being analyzed by the same laboratory or method. As this is impractical in many cases, it is of great importance that the laboratory as well as the assays used comply with the current recommendations regarding GH measurement ( 4 ). The laboratory should also participate in external quality assessment schemes, where aliquots of the same samples are distributed to many laboratories for blinded assessment of GH concentration. Comparison of the results allows understanding the relative bias of GH concentrations reported by the local laboratory to those reported by other laboratories measuring GH. Results of such external quality assessment schemes are publicly available (e.g. https://www.rfb.bio/). As shown in Figure 1 , concentrations for the two samples vary widely between labs ( 1 A), but are consistently higher or lower in laboratories using the same analytical method ( 1 B). Such data can be very useful for the clinician when trying to adapt information from the literature to GH concentrations obtained by the local GH assay.

### Biological variables in the interpretation of measured GH concentrations

The analytical variability introduced by the different assay methods adds to the huge biological variability. GH secretion is influenced by anthropometric, metabolic and pharmacologic factors. While some obvious cases of GHD or acromegaly might be easy to interpret, awareness of such confounding factors can be crucial in borderline cases, or when concentrations of GH and IGF-I are discordant. BMI is a major determinant of GH secretion. Obese individuals exhibit significantly lower GH concentrations at baseline and during many stimulation tests, but also exhibit more pronounced suppression of GH secretion during OGTT ( 26 - 28 ). Furthermore, factors such as stress, sleep, nutritional status, exercise and sex hormones influence GH secretion ( Table 2 ). Females on oral estrogens can exhibit greatly reduced suppression of GH during OGTT. Retesting after pausing medication may be considered if GH concentration remain with no clinical signs of acromegaly ( 26 ). A complex biological confounder of GH stimulation tests is pubertal development. There is dissent among experts and guidelines, but some recommend priming with sex steroids beyond a certain age to enable standardized, age-adjusted GH secretion ( 5 , 23 ).

**Table 2:** Factors influencing GH and IGF-I concentration

Influencing factor	GH	IGF-I	Remarks	References (examples)
Obesity	↓	↔ ↓	Decreased GH secretion (possibly through low ghrelin concentrations). Normal IGF-I bioactivity.	( 26 , 28 )
Fasting	↑	↓	Decreased metabolic clearance of GH, hepatic GH resistance.	( 55 , 81 )
Malnutrition	↑	↓	Decreased IGF-I secretion, lack of IGF-I feedback and, thus, increased GH secretion.	( 56 )
Anorexia nervosa	↑	↓	Hepatic GH-resistance, reduced IGF-I bioactivity.	( 28 )
Stress (acute)	↑	↔	Stimulation of GH secretion.	( 82 )
Exercise	↑	↔	Stimulation of GH secretion.	( 82 )
Sleep	↑	↔	Increased GH secretion in slow-wave sleep (deep sleep).	( 83 )
Glucose intake	↓	↔	Decreased GH secretion.	( 26 )
Type 1 Diabetes mellitus	↑	↔ ↓	Hepatic GH-resistance.	( 57 )
Type 2 diabetes mellitus	↓	↔ ↓	Hepatic GH-resistance.	( 58 )
Chronic renal failure and uremia	↔ ↑	↔ ↓	Reduced renal GH degradation and GH resistance. IGF-I bioactivity is reduced due to elevated binding protein concentrations.	( 59 )
Liver disease	↑	↓	Reduced IGF-I production, increased GH secretion through negative feedback mechanism and hepatic GH resistance.	( 61 )
Hypothyroidism	↓	↓	Decreased GH secretion in long term hypothyroidism.	( 64 )
Hyperthyroidism	↓	↑	Decreased GH secretion. Increased IGF binding proteins and therefore reduced IGF-I bioactivity.	( 64 )
Acute critical illness	↑	↓	Hepatic GH-resistance.	( 62 )
Systemic inflammation	↑	↓	Hepatic GH-resistance.	( 63 )
Oral estrogens	↑	↓	Hepatic GH-resistance.	( 26 )
Testosterone	↔ (↑)	↑	No changes in long-term therapy (> 6 weeks).	( 65 )
Biotin intake*	↓	↓	depends on susceptibility of specific assay used, GH and IGF-I can be falsely low.	Personal observation (unpublished)
Pegvisomant	↑ ↓ ↔	↓ **	Influence of pegvisomant on GH dependent on assay used (see Table 1 ).	( 11 )
Pregnancy	↑ ↔	↑	GH cross reactivity dependent on assay used (see Table 1 ).	( 11 )

GH: growth hormone; IGF-I: insulin-like growth factor I; ↓ decreased; ↔ unchanged; ↑ increased. *Applies only, if biotin-streptavidin system is used. **Therapeutically desired effect.

### Unexpected interference in GH measurements

Occasionally, factors, which on the first instance seem unrelated to hormone measurements, can heavily affect the analytical process. One example is the increasing use of dietary supplements by patients. Many of these supplements contain – among other vitamins and trace elements – significant amounts of biotin. Unfortunately, most of today’s immunoassays for hormone measurements also use biotin (together with streptavidin) for signal generation and amplification. Since the biotin intake in users of dietary supplements can lead to significant amounts of free biotin in blood samples, this can interfere with signal generation in the immunoassays. Depending on assay design, measured hormone concentrations become falsely high or falsely low. In sandwich type immunoassays, which are routinely used for GH (and IGF-I) measurements, biotin can lead to falsely low concentrations. Physicians should be aware of this potential source of interference when seeing implausibly low results for GH (and IGF-I) and ask their patients about recent use of nutritional supplements. An efficient and easy measure to overcome the biotin interference is stopping biotin intake 24 hours before drawing the blood sample.

Another situation where interpretation of GH concentrations can be problematic is in pregnant females: Physiologically, the syncytiotrophoblast of the placenta takes a lead in secreting a variant of GH, named GH-V. In turn, pituitary GH secretion is suppressed through the increase in IGF-I, induced by high tonic GH-V secretion. Depending on the antibody used in the local GH assay, cross-reactivity can lead to falsely high GH concentrations. If there is a need to asses GH secretory status during pregnancy, it is mandatory to use an assay without cross-reactivity with GH-V ( 11 ). Notably, it is also possible to measure GH-V without cross-reactivity from pituitary GH, though the physiological relevance of GH-V remains to be elucidated ( 29 ).

Finally, in patients who are treated with the GH antagonist pegvisomant, GH concentrations, as measured by most commercially available assays, are unreliable. Depending on the binding site of the antibody, pegvisomant can lead to falsely high or falsely low GH concentrations. Based on published information, there is only one commercially available GH assay that does not cross-react with pegvisomant and therefore can be used to investigate GH secretory status in the respective patients on treatment ( 11 ).

### 20 kDa GH isoform

The 20kDa GH isoform does not play a role in routine diagnostics, and there is no assay to measure 20kD GH which is commercially available. However, assays specific for 20kD GH have been established in research labs. Some groups have reported that the 20/22kDa ratio is elevated in patients with acromegaly. Furthermore, it has been shown that 20kD GH decreases after injection of recombinant 22kD GH, making a 20kD GH assay potentially useful in doping analyses ( 30 , 31 ).

## IGF-I

### IGF-I molecule

Some of the effects of GH are direct effects, but the majority is mediated via IGF-I. IGF-I is a smaller polypeptide hormone (molecular weight 7.66 kD) that shares structural similarities with insulin. It is synthesized in many tissues but mainly in the liver. Binding of GH to the pre-dimerized GH receptor induces a conformational change which triggers intracellular signaling and, subsequently, IGF-I synthesis and release ( 32 ). In contrast to the pulsatile secretion of GH, IGF-I is more constant, and does not show significant circadian variation. Its close relationship with GH secretion and its stability make IGF-I a good surrogate marker of GH action in clinical practice.

While rather stable during shorter time-periods, IGF-I exhibits a strong age-related secretion pattern: Concentrations decline immediately after birth, but start to increase after the first year of life ( 33 ). The peak is reached at puberty. Later in life, concentrations steadily decline. In serum, 95% of IGF-I is bound to a family of IGF binding proteins (IGFBPs, IGFBP- 1 through IGFBP- 7 ). Among those binding proteins, IGFBP-3 is the most abundant and most important one ( 34 ). In circulation, IGF-I together with the binding proteins-3 and -5 form a ternary complex together with another liver-derived molecule named “acid labile subunit” (ALS). The resulting ternary complex has a molecular weight of approx. 150kDa. Complex formation significantly prolongs the half-life of IGF-I ( 35 , 36 ).

### Technical aspects of IGF-I measurement

**Figure 2:**
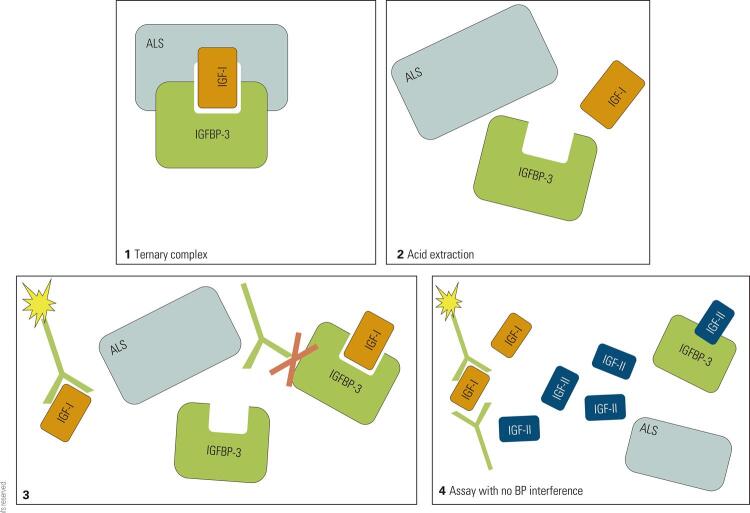
IGF-I immunoassay scheme. The ternary complex ( 1 ) is dissociated by acid extraction ( 2 ), but re-aggregation of the components during incubation can impair antibody binding to IGF-I ( 3 ). Addition of excess IGF-II blocks binding protein interference ( 4 ). IGF-I: insulin-like growth factor I; IGF-II: insulin-like growth factor II; IGFBP-3: insulin-like growth factor binding protein-3; ALS: acid labile subunit; BP: binding protein.

The first radioimmunoassays for IGF-I (somatomedin-C) were developed in the 1970^th^ ( 37 ). Similar to the evolution of GH assays, also assays to measure IGF-I evolved from polyclonal radioimmunoassays to monoclonal automated chemiluminescence immunoassays. As for GH, heterogeneity of the different assays also represents a problem in comparability of IGF-I concentrations obtained by different methods ( 38 ). The main reasons for the heterogeneity are the use of different IGF-I calibration standards (National Institute for Biological Standards and Control (NIBSC) 87/517 or 02/254), the use of different antibodies with different epitope specificities and binding affinities, and the potential interference from binding proteins ( 4 , 39 ). As one step forward to harmonization of IGF-I assays, the Growth Hormone Research Society and the International Society for IGF Research recommended to uniformly use the latest, recombinant and pure reference preparation for calibration (WHO NIBSC 02/254) for all IGF-I assays. Following this request from 2011, several of the assay manufacturers reacted. Currently, all of the automated IGF-I assays and many of the manual assays claim to be calibrated against this standard ( Table 1 ) ( 4 ). Nonetheless, IGF-I measurement with different assays still yields in different IGF-I concentrations ( 38 , 40 ). A particular technical challenge when measuring IGF-I is the removal of binding protein interference. To make the IGF-I molecule accessible to the antibodies during the measurement procedure, IGF-I has to be liberated from the ternary complex. This can be achieved by different extraction procedures (i.e. acid extraction) ( 41 ). However, fast re-aggregation of IGF-I and its binding proteins must be prevented during the incubation step with the specific antibodies, and in this step commercially available assays differ significantly. A gold-standard method ( 42 ) to keep the molecules separated is the addition of excess IGF-II ( Figure 2 ), which is effective, yet costly. More recently, some groups have established IGF-I measurements based on liquid-chromatography/mass-spectrometry. While associated with some theoretical advances in terms of specificity, several of the problems associated with immunoassay measurement of IGF-I also seem to affect comparability of IGF-I measurements from different laboratories using different LC-MS/MS based methods. The methods differ with respect to the protocols used to digest samples, in the instruments and measurement specifications used, but also in the standard preparations used. Therefore, it is no surprise that different LC-MS/MS assays for IGF-I do not agree any better than different immunoassays ( 43 , 44 ). Currently, the better-validated LC-MS/MS based assays seem to be as reliable as the better immunoassays, and both analytical platforms seem to allow establishing assays that exhibit good correlation among each other ( 45 ). For the clinician, it is important to be aware of the methodological differences between assays. As for GH, it would be ideal if the same IGF-I assay would be used in follow-up of a patient with GHD or acromegaly. However, laboratories should at least participate in external quality assessment schemes and know the relative bias of their IGF-I assay to other methods to assist clinicians when confronted with IGF-I results from other laboratories.

### IGF-I reference intervals

The consensus statement from 2011 established basic requirements for IGF-I reference intervals ( 4 ):

1. They must be assay specific (i.e. specific to the assay method used by the respective laboratory).
2. They must be established based on transparently described, well-characterized large cohorts representing the “normal” background population, and include an appropriate number of subjects of all age groups and from both sexes.
3. The lower and upper limit of normal (2.5^th^ and 97.5^th^ percentile) have to be reported in mass units, and an approach to convert IGF-I concentrations to standard deviation scores (SDS) or z scores must be provided.

Only very few published studies on reference intervals for IGF-I fulfil all the criteria, and significant differences exist with respect to e.g. cohort size or statistical method employed. To define sex- and age-adjusted reference intervals, different studies used log transformation, polynomial modelling, regression analysis (linear, quantile), parametric method or the LMS approach ( 33 , 38 , 46 - 52 ).

For adults, reference ranges should be at least decade-specific, but in children smaller increments in age groups are mandatory. During adolescence, it can be beneficial to use reference ranges specifically adjusted for Tanner stages ( 33 , 51 ). Because the chronological age at onset of puberty as well as the speed of progression exhibit great variability, the lower limit of the reference interval (2.5^th^ percentile) is significantly higher in both sexes, and the IGF-I peak occurs earlier in girls, if evaluated according to Tanner stages rather than chronological age ( 33 , 51 ).

Most problems occur if different IGF-I assays, together with reference intervals inappropriate for the actual assay method, are used during monitoring of a single patient. However, we must accept that, even if the same assay and the same statistical methods are used, reference intervals of different origin still will be different, depending on cohort size, age range, inclusion criteria or true differences in characteristics of the background population ( 33 , 38 ). Such differences can be significant, change the interpretation, and lead to differences in diagnosis, initiation of treatment or long-term patient management ( 33 , 50 , 53 ).

### Biological variables affecting IGF-I concentrations

Although mainly stimulated by GH, IGF-I concentrations are also influenced by a variety of other factors ( Table 2 ). For example, BMI and nutritional status are important determinants: IGF-I tends to be lower in morbid obesity, but can be increased with weight loss ( 28 , 54 ). On the other extreme, in prolonged fasting, anorexia nervosa and malnutrition, IGF-I is also reduced ( 28 , 55 , 56 ). The same can be found in patients with type 1 or type 2 diabetes mellitus, depending on diabetes control ( 57 , 58 ). Lower IGF-I has been reported in the majority of cases of chronic kidney disease and uremia, but most importantly, in these conditions its bioactivity is reduced, perhaps due to impaired elimination of binding proteins ( 59 , 60 ). In liver diseases, depending of the severity of the disease, IGF-I synthesis may be impaired, resulting in lower IGF-I concentrations ( 61 ). Furthermore, reduced expression of GHR and GH resistance can occur, a mechanism which also has been suggested to explain the reduced IGF-I concentrations seen in acute critical illness and systemic inflammation ( 62 , 63 ). Hypo- and hyperthyroidism affect IGF-I concentrations, which are positively correlated to fT3 concentrations. Appropriate treatment normalizes IGF-I in thyroid disease ( 64 ). Due to estrogen-induced hepatic GH resistance, females on oral estrogens require more GH to achieve the same concentration of IGF-I. Accordingly, IGF-I on oral estrogens is lower compared to premenopausal women without any hormonal contraception, but also to women with transdermal estrogen application or gestagen monotherapy ( 26 ). Testosterone replacement, on the contrary, has been shown to increase IGF-I concentrations ( 65 ).

Sensitivity and specificity of IGF-I as a biomarker for GH related diseases varies with age, even when age-adjusted reference intervals are available. Some authors reported poor sensitivity of IGF-I for diagnosis of GHD in younger children (< 3 years) and proposed IGFBP-3 as a superior maker in that age group ( 66 ). This observation might be explained by the fact that for many IGF-I assays, the lower end of the reference interval in younger children overlaps with the limit of quantification of the assay. Accordingly, a recent consensus on diagnosis of GHD in children emphasized assay sensitivity as a critical quality criterion ( 23 ). Beyond the age of 50, IGF-I concentrations can be low in healthy subjects and overlap with concentrations seen in GHD patients. Therefore, IGF-I is less specific as a diagnostic marker for GHD in this age group ( 67 ). In acromegaly, sensitivity and specificity of IGF-I are relatively high throughout life. However, limitations exist at higher GH concentrations (> 10 g/L), where IGF-I exhibits a ceiling effect with no further increase proportional to the increase in GH. This must be taken into account in acromegaly, where drastic reductions in GH secretion initially might be accompanied by only relatively small reductions in IGF-I.

### Free IGF-I

IGF-I assays used in clinical routine measure total IGF-I, which in most cases is reasonable to assess the GH-IGF-I axis. Only in specific clinical conditions where concentrations of binding protein concentrations can be significantly altered (i.e. chronic renal failure, starvation), total IGF-I might no longer be the most reliable biomarker ( 60 ). In such cases, measurement of free IGF-I (or bioactive IGF-I) has been shown to provide additional information ( 68 ). This is also true for rare cases of patients with short stature caused by mutations in pregnancy-associated plasma protein A2 (PAPP-A 2 ), as liberation of IGF-I from IGFBP-3 and -5 by the metalloproteinase PAPP-A2 is impaired in these patients ( 69 ). Direct measurement of “free IGF-I” requires sophisticated analytical methods which are available in very few laboratories only. As an easier available surrogate marker, the IGF-I/IGFBP-3 molar ratio has been suggested. It might not provide all the information which could be extracted from direct assessment of free IGF-I, but has been shown to correlate with clinical endpoints such as efficacy of recombinant human GH treatment ( 70 , 71 ).

## IGFBP 3

IGFBP 3 is the most abundant IGF binding protein and – since its synthesis is also stimulated by GH - is used itself as a biomarker of integrated GH secretion ( 72 ). The age-related pattern of IGFBP 3 concentrations largely mirrors that of IGF-I, though the decline in adults is less obvious. IGFBP 3 concentrations have been reported to be decreased in liver disease and fasting, but increased in chronic renal failure due to impaired excretion ( 72 ).

As mentioned above, some advantage has been demonstrated especially in in young children (<3 years), where IGFBP 3 correlates well with integrated GH secretion, and seems more sensitive than IGF-I in the diagnosis of GHD ( 66 , 73 ). This advantage of IGFBP 3, however, might be a consequence of shortcomings of the IGF-I assays used rather than an inherent advantage of IGFBP 3. In patients with acromegaly, correlation with GH secretion and IGF-I concentrations is also fairly good ( 72 ), but overall, the diagnostic value of IGFBP 3 seems limited. In most circumstances, it cannot add additional information beyond IGF-I. If measurement of IGFBP 3 is considered, however, the same criteria for quality of the reference intervals as for IGF-I must be applied: Such reference intervals must be assay-, age- and sex-specific, and reference intervals adjusted to Tanner stages might be required in adolescents, ( 74 ).

## OTHER GH-RELATED PARAMETERS

### ALS

ALS is another protein which is synthesized in a GH-dependent manner in the liver, and which in circulation becomes part of the ternary complex with high affinity for the binary IGF-I/IGFBP 3 complex ( 35 ) ( Figure 2 ). It has been shown to correlate with IGF-I in GHD as well as in acromegaly, but its measurement seems not to add significant information to measurements of IGF-I or IGFBP 3, respectively ( 36 ). ALS measurements are technically challenging due to large leucine rich repeats in the molecule. To quantify the molecule, antibodies with particular specificity and – according to some protocols – pretreatment procedures are required. Interestingly, some pretreatment procedures of the serum sample seem to be associated with more clinical relevance ( 75 ). A very rare indication to measure ALS can be the suspicion of a deletion in the ALS gene, a disease associated with severe GHD. In these patients, circulating ALS concentrations are very low or undetectable ( 76 ).

### Klotho

Alpha soluble klotho is mainly expressed in the kidney and the choroid plexus, but to a lesser extend also in the pituitary gland ( 77 ). It has recently been discovered that soluble alpha klotho positively correlates with IGF-I and GH in acromegaly ( 78 ). After successful surgery as well as with treatment with first-generation SSA, αKL decreases ( 78 , 79 ). Notably, and in contrast to IGF-I, αKL correlates very well even with markedly elevated GH concentrations (> 10 µg/L). This might be of particular importance in patients with newly diagnosed or not successfully operated patients with uncontrolled acromegaly and GH concentrations > 10 µg/L: As mentioned above, in that range of high GH concentrations, IGF-I has been shown to exhibit a ceiling effect, making it less reliable as a biomarker for dose titration of any administered drug. Another potential advantage of αKL, compared to IGF-I and GH, might be that it is not BMI and sex-dependent ( 79 ). This might facilitate interpretation of αKL concentrations compared to those of IGF-I. Potentially, αKL might be relevant in the investigation of patients with discordant GH and IGF-I concentrations. However, more studies are required to define the clinical utility of this new biomarker of GH secretion.

### GHBP

Approximately half of the circulating GH is bound to GHBP, which resembles the extracellular domain of the GHR ( 80 ). In cases of short stature with growth hormone insensitivity, it is diagnostically relevant, as it might differentiate between GHD and GH insensitivity, for example in GHR defects such as Laron’s syndrome, where GHPB is very low or undetectable. However, there are also genetic defects of the GHR with normal or even elevated GHBP ( 80 ). As with other GH-related parameters, GHBP concentrations are influenced by age, sex, body composition and oral estrogen intake, which has to be taken into account in evaluation. Assays to measure GHBP are less widely available, and caution must be applied to interpret the results in view of appropriate reference intervals.

## CONCLUSIONS

Measurements of different biomarkers have been proposed for the diagnosis of diseases related to the GH-IGF-I axis, as well as for monitoring of therapy. Currently, IGF-I is considered the most important and reliable surrogate parameter reflecting GH secretory status, and its measurement is recommended in the guidelines for acromegaly and GHD. Measurement of integrated, stimulated or suppressed GH is used as the most important confirmatory parameter. Measuring GH and IGF-I in blood samples is not without pitfalls, and laboratories are requested to only use methods which have been proven to adhere to recent guidelines. Calibration, removal of binding protein interference, long-term stability of assay performance and quality of method-specific reference intervals or cut-offs are critical aspects of GH and IGF-I assay quality. Obviously, meaningful interpretation of data is also possible only when the biological variability and a wide variety of potential confounders of both parameters are taken into account. Determination of other parameters such as IGFBP-3, ALS, alpha soluble klotho and GHBP have not been shown to add significant information in the majority of cases. However, their measurement might be useful in certain age groups, in diagnostically ambiguous cases or in the diagnostic workup of rare genetic diseases.
